# Exploring the role of social capital in enhancing physical activity among college and university students: A systematic review

**DOI:** 10.1371/journal.pone.0314610

**Published:** 2024-11-27

**Authors:** Zhendong Gao, Chen Soon Chee, Roxana Dev Omar Dev, Fangyi Li, Rui Li, Jianhong Gao, Yutong Liu

**Affiliations:** 1 Faculty of Educational Studies, Department of Sports Studies, Universiti Putra Malaysia, Serdang, Malaysia; 2 Department of Sports Teaching and Research, Lanzhou University, Lanzhou, China; University of Glasgow, UNITED KINGDOM OF GREAT BRITAIN AND NORTHERN IRELAND

## Abstract

College and university students often exhibit insufficient levels of physical activity, which negatively impacts their health, mental well-being, and academic performance. Social capital has emerged as a potential factor in promoting physical activity. This systematic review, conducted in line with PRISMA guidelines, examines the existing literature on the role of social capital in enhancing physical activity among college and university students. A search of the Web of Science, Scopus, SportDiscus, and PsychINFO databases identified 10 studies published by August 1, 2024, involving 2,700 students, primarily from North America and Europe. The overall quality of the included studies was high, with all scoring above 85% on quality assessments. The findings indicate that social capital, particularly strong social networks, support from family and friends, and high levels of social cohesion, is significantly associated with higher levels of physical activity among college and university students. Social capital may facilitate physical activity in group settings through mechanisms such as emotional support, role modeling, and social norms. Despite limitations within the existing research evidence, such as reliance on self-reported data and cross-sectional study designs, this review suggests that social capital holds potential for promoting physical activity in this population. Future research should prioritize the use of objective measurement tools and longitudinal designs to more accurately assess the long-term effects of social capital and explore how these findings can inform effective interventions.

## 1. Introduction

Physical activity is widely regarded as a key component in maintaining physical health, mental well-being, and overall quality of life [1,2]. Across the lifespan, regular participation in physical activity has been shown to reduce the risk of chronic diseases, enhance cognitive function, and improve mental health outcomes [3–5]. Given the broad impact of physical activity at different stages of life, promoting physical activity remains a central priority in public health.

Globally, college and university students commonly exhibit insufficient levels of physical activity, making this group a particular focus of public health concern [6–8]. While physical activity is important at all stages of life, research suggests that if physical activity levels are low in early adulthood, it is less likely to become a regular part of one’s lifestyle later on [9]. Moreover, the higher education period represents a critical phase for establishing and consolidating healthy lifestyle habits [10–12]. The reasons for insufficient physical activity among college and university students are multifaceted, involving individual, environmental, and social factors [13,14]. For many, the transition from high school to college or university represents a key turning point in life, often accompanied by significant lifestyle changes, which frequently lead to an overall decline in physical activity levels [15–17]. A systematic review and meta-analysis on physical activity during educational transitions in early adulthood found that leaving high school correlated with a daily decrease of −7.04 minutes (95% CI, −11.26 to −2.82) in moderate-to-vigorous physical activity [18]. Another similar systematic review found that physical activity levels moderately decline as individuals transition from adolescence to early adulthood, with a daily decrease of 3.4 minutes of moderate-to-vigorous physical activity (WMD -5.2 to -7.3 minutes) [19]. Additionally, a systematic review of barriers to physical activity among high school and university students identified key obstacles for undergraduates, including psychological, emotional, and cognitive factors (e.g., lack of time and motivation), environmental barriers (e.g., lack of accessible spaces), and socio-economic and demographic factors (e.g., lack of financial resources) [20]. A separate systematic review on key factors influencing physical activity among university students highlighted the significance of environmental context, social influence, and goal-oriented factors within theoretical frameworks, as identified in the majority of studies [21].

In recent years, researchers have proposed various strategies to promote physical activity, such as improving campus sports facilities, introducing health education programs, and utilizing digital health applications [6,8,22]. Among these strategies, interventions aimed at enhancing social capital have garnered significant attention [23–25]. The core elements of social capital include social networks, social participation, trust, and reciprocity. Although the definition and measurement of social capital remain subjects of debate in academic circles, these elements are widely recognized [26,27]. Generally, social capital can be conceptualized as the social resources individuals and groups acquire through social networks, trust, and reciprocal relationships [28]. Its key dimensions include individual-level attributes such as the quality and quantity of social networks, social support, and information channels (focused on network/support measures); and collective-level attributes such as the degree of mutual trust among community members, and shared social norms and values (focused on social cohesion measures) [28,29].

Specifically, social networks refer to the structure and patterns of relationships between individuals and their social connections [30]. These networks reflect the breadth and depth of connections individuals have with family members, friends, classmates, colleagues, and community members. The quality and quantity of social networks significantly influence the social resources, support systems, and flow of information that individuals can access [31]. Meanwhile, social support refers to the emotional, instrumental, and informational assistance that individuals receive through their social networks [32]. Social support focuses on the cognitive/functional qualitative aspects of interpersonal relationships, such as the content and availability of relationships with significant others, while social networks focus on the quantitative and structural aspects of these relationships [32]. These network relationships can be assessed at both the individual and group levels and can be conceptualized and measured as constructs at either level [29]. Social cohesion, on the other hand, reflects the solidarity, norms, trust, and reciprocity among members of a group or community [33]. It encompasses the degree of trust community members have in one another, shared goals, and adherence to common social norms and values. This can be understood as a contextual or collective attribute, emphasizing the "contextual" influence that the collective exerts on the individual [29].

Current research indicates that interventions aimed at promoting social capital can significantly influence health behaviors at both individual and community levels [34,35]. In the context of college and university students, these interventions may indirectly promote physical activity in various ways. For example, enhancing social support from family and friends can increase motivation to engage in physical activity [36]; expanding social networks can provide resources and opportunities for participation in physical activities [37]; and encouraging active social participation can create a social environment that promotes healthy lifestyles, thereby fostering physical activity [38].

However, to the best of our knowledge, while several studies have explored the relationship between social capital and physical activity [39–42], the specific mechanisms and underlying connections in college and university students remain relatively under-researched. Furthermore, existing reviews on the association between social capital and physical activity primarily focus on network/support-based measures of social capital [43–45], with limited attention to measures based on social cohesion. Therefore, this study aims to conduct a systematic review of the existing literature, integrating both qualitative and quantitative findings to analyze the role of social capital in promoting physical activity among college and university students. Through this review, we hope to identify gaps and unresolved issues in the current body of research and provide a more comprehensive and coherent perspective. Our goal is to offer valuable insights to policymakers, educators, and public health practitioners, helping them better understand the complex relationship between social capital and physical activity among college and university students. These insights will contribute to the design of more effective interventions to enhance physical activity levels and, ultimately, improve the overall health of this population.

## 2. Materials and methods

This systematic review was conducted following the guidelines provided in the PRISMA statement for systematic reviews and meta-analyses [46] (see S1 Checklist).

### 2.1. Search strategy

Based on previous systematic reviews on related topics [34,35,47], we conducted a literature search in August 2024 using the Web of Science, Scopus, SportDiscus, and PsychINFO databases, with the search period ending on August 1, 2024. These databases were selected for their high credibility and widespread recognition in the fields of public health and sports science. We used a combination of search terms including “social capital,” “physical activity,” and “college and university students.” The detailed search strategy is outlined in Table 1. Abstracts, conference proceedings, theses, book chapters, and articles published in non-peer-reviewed journals were excluded from the search. Additionally, the reference lists of included studies and prior review articles were checked for relevant citations.

**Table 1:** Search strategy.

Type of database	Searching type	Result
Web of Science	TS = ("social capital" OR "social cohesion" OR "collective efficacy" OR "social trust" OR "social networks" OR "social engagement" OR "social participation" OR "social integration" OR "social relationships" OR "social ties" OR "reciprocity" OR "social connections" OR "social connectedness") AND TS = ("physical activity" OR "motor activity" OR "physical exertion" OR "sports" OR "exercise" OR "leisure physical activities" OR "leisure activities" OR "physical exercise" OR "physical inactivity") AND TS = ("College" OR "Colleges" OR "College student" OR "College students" OR "Higher education" OR "Undergraduate" OR "Undergraduates" OR "University" OR "Universities") AND DT = (article) AND LA = (English)	211
Scopus	(TITLE-ABS-KEY ("social capital" OR "social cohesion" OR "collective efficacy" OR "social trust" OR "social networks" OR "social engagement" "social participation" OR "social integration" OR "social relationships" OR "social ties" OR "reciprocity" OR "social connections" OR "social connectedness") AND TITLE-ABS-KEY ("physical activity" OR "motor activity" OR "physical exertion" OR "sports" OR "exercise" OR "leisure physical activities" OR "leisure activities" OR "physical exercise" OR "physical inactivity") AND TITLE-ABS-KEY ("College" OR "Colleges" OR "College student" OR "College students" OR "Higher education" OR "Undergraduate" OR "Undergraduates" OR "University" OR "Universities") ) AND (LIMIT-TO (DOCTYPE, "ar") )	34
SportDiscus	AB ("social capital" OR "social cohesion" OR "collective efficacy" OR "social trust" OR "social networks" OR "social engagement" "social participation" OR "social integration" OR "social relationships" OR "social ties" OR "reciprocity" OR "social connections" OR "social connectedness") AND AB ("physical activity" OR "motor activity" OR "physical exertion" OR "sports" OR "exercise" OR "leisure physical activities" OR "leisure activities" OR "physical exercise" OR "physical inactivity") AND AB ("College" OR "Colleges" OR "College student" OR "College students" OR "Higher education" OR "Undergraduate" OR "Undergraduates" OR "University" OR "Universities")	55
PsychINFO	"AB ("social capital" OR "social cohesion" OR "collective efficacy" OR "social trust" OR "social networks" OR "social engagement" "social participation" OR "social integration" OR "social relationships" OR "social ties" OR "reciprocity" OR "social connections" OR "social connectedness") AND AB ("physical activity" OR "motor activity" OR "physical exertion" OR "sports" OR "exercise" OR "leisure physical activities" OR "leisure activities" OR "physical exercise" OR "physical inactivity") AND AB ("College" OR "Colleges" OR "College student" OR "College students" OR "Higher education" OR "Undergraduate" OR "Undergraduates" OR "University" OR "Universities")	71

### 2.2. Inclusion/Exclusion criteria

The inclusion criteria were as follows: 1) studies must be related to at least one distinct group of healthy college or university students; 2) studies that examined the relationship between social capital and physical activity, including both qualitative and quantitative research; 3) inclusion of at least one social capital indicator; 4) provision of at least one result related to the association between social capital and physical activity (quantitative: e.g., correlations; qualitative: e.g., categories); 5) articles published in peer-reviewed journals in English by August 1, 2024. These criteria were selected to ensure that the studies reviewed are directly relevant to the college and university student population and provide meaningful insights into the relationship between social capital and physical activity. By including both qualitative and quantitative studies, we aimed to capture a broad range of evidence reflecting various aspects of social capital. We focused on peer-reviewed English-language articles to ensure the quality and accessibility of the studies reviewed.

The exclusion criteria were: 1) studies not involving college or university student populations; 2) studies that did not provide results linking social capital measures to physical activity measures; 3) reviews, opinion articles, or theoretical papers; 4) studies for which full texts were not available or data were incomplete; 5) studies that only included measures of social support. The exclusion criteria were designed to filter out studies that were not directly relevant to our research focus. Studies with incomplete data or those that did not establish a connection between social capital and physical activity were also excluded to maintain the rigor and relevance of the review. Additionally, studies focusing solely on social support were excluded, as there are already reviews addressing the relationship between social support and physical activity among college and university students [44,48]. We focused on understanding social capital through the lens of social cohesion, given that the search for social capital inevitably includes approaches based on both social support and social cohesion [34,49].

### 2.3. Screening of retrieved citations

The screening process involved a tiered evaluation. Initially, citations retrieved from the search were downloaded into Endnote X7 after removing duplicates, to facilitate study selection based on titles and abstracts. Subsequently, the full texts of the remaining studies were retrieved and assessed for eligibility. If necessary, both abstracts and full texts were further screened. The search and selection process was independently conducted by four researchers (GZ, LR, LF, GJ). Any discrepancies regarding the inclusion of specific studies were resolved through consensus meetings. If consensus could not be reached, final decisions on inclusion or exclusion were made by two additional researchers (CC and RD). Basic information for each retrieved article (i.e., author, publication year, and article title) was recorded by one author (GZ) in a Microsoft Excel® spreadsheet to ensure comprehensive audit tracking (see S1 File).

### 2.4. Data extraction and quality assessment

The data extraction process was independently conducted by two reviewers (GZ, LY). In cases of discrepancies, the reviewers (CC and RD) were consulted, and any differences were resolved through consensus. Key elements from each study were extracted and summarized in a table, listed chronologically by publication year. The table for each study included the following information: the first author and publication year, sample details (size, characteristics, location), study design (quantitative/qualitative, cross-sectional, prospective, or experimental), social capital measures, physical activity measures, covariates included, and main findings.

Each article was evaluated for quality using the criteria proposed by Kmet et al. [50]. Both qualitative and quantitative articles were assessed using the corresponding checklists. For quantitative studies, a 14-item checklist was used to score each article based on how well it met each criterion (2 = fully meets the criterion, 1 = partially meets the criterion, 0 = does not meet the criterion). Items not relevant to the specific study objectives were marked as “n/a.” The quality of qualitative studies was assessed using a 10-item checklist, with the same scoring system as for quantitative articles. An overall quality score for each article was calculated based on the relevant criteria and then standardized into a percentage. Scores of ≤55%, 55–75%, and ≥75% were considered low, moderate, and high quality, respectively [50]. All included quantitative and qualitative studies and their respective quality scores were independently assessed by the author (LF) and then double-screened by the author (LY) to confirm the appropriateness of the results and ensure consistency in quality assessment.

### 2.5 Data analysis

A meta-analysis was not conducted due to significant heterogeneity across the included studies. The quantitative studies varied considerably in terms of research design, participant characteristics, and, most notably, the measurement of social capital. These differences made statistical synthesis inappropriate. Additionally, one qualitative study was included, which provided valuable contextual insights but could not be quantitatively synthesized. As a result, a narrative synthesis was conducted to integrate findings from both the quantitative and qualitative studies, allowing for a broader understanding of the relationship between social capital and physical activity among university students.

## 3. Results

### 3.1. Study selection

**Fig 1:**
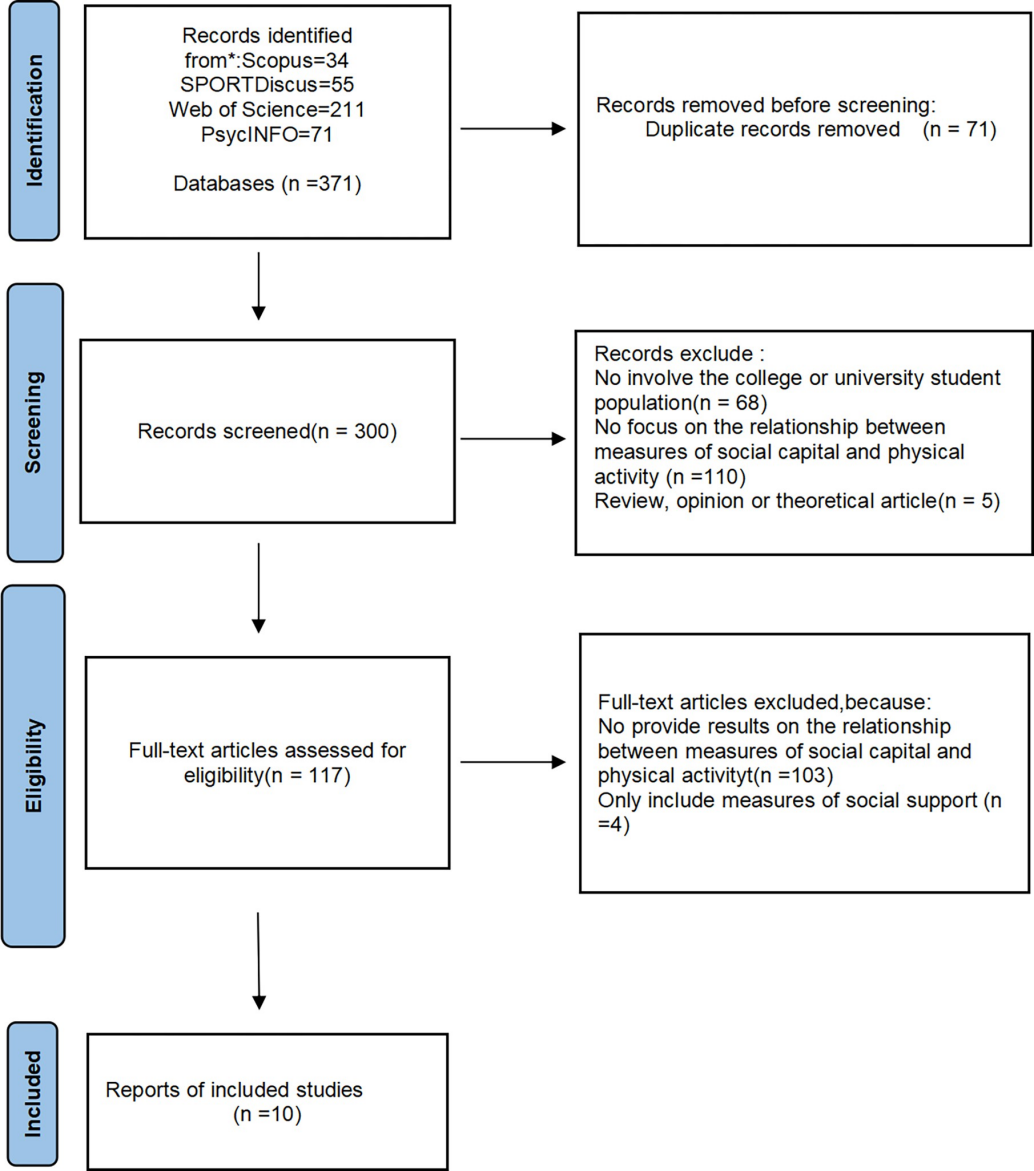
PRISMA flowchart showing how the final sample of 10 studies was obtained (applying inclusion/exclusion criteria).

As shown in Fig 1, the initial search retrieved 371 published papers. After removing 71 duplicate records, 300 papers were screened based on their titles and abstracts. During the screening phase, 68 records were excluded because they did not involve college or university student populations. An additional 110 records were excluded because they did not examine the relationship between social capital measures and physical activity measures. Five more records were excluded because they were reviews, opinion articles, or theoretical papers rather than empirical studies. Following this, the full texts of 117 articles were assessed for eligibility. After full-text review, 103 articles were excluded because they did not provide results linking social capital measures to physical activity measures, 4 articles were excluded for focusing solely on social support measures. Ultimately, 10 studies met all inclusion criteria and were included in this systematic review.

### 3.2. Study characteristics

Table 2 summarizes the characteristics of the 10 studies that met the inclusion criteria for this review. The final sample included a variety of study designs, comprising 7 quantitative cross-sectional studies, 2 quantitative longitudinal studies, and 1 qualitative study. These studies collectively involved 2,700 college and university students. Participant ages ranged from 17 to 61 years, with most studies focusing on young adults aged 18–24 years. The gender distribution was predominantly female, with the proportion of female participants ranging from 54% to 82.7%. Participants came from diverse cultural backgrounds, though most studies were conducted in Western contexts, primarily in North America and Europe. Specifically, six studies were conducted in the United States, two in Canada, one in Belgium, and one in Germany. The majority of participants were from White and Western backgrounds, although some studies included participants from various cultural and ethnic backgrounds. Most (8) of the included studies focused on social capital measures related to networks and support, while a smaller portion (2) addressed social capital measures related to social cohesion. Physical activity was measured using various self-report methods. Common indicators included the frequency and duration of moderate to vigorous physical activity (MVPA), adherence to physical activity recommendations (e.g., 150 minutes of MVPA per week), and self-reported weekly exercise time.

**Table 2:** Results of literature review.

Author/Year	Study Design	Participant characteristics (age [mean and range], gender)	Location	Cultural background	Social capital indicator	Physical activity indicators	Covariates	Result
(Barnett et al., 2014) [51]	Cross-sectional	N = 129 undergraduates (62F/67M,AR≥18)	Northeastern, America	Predominantly non-Hispanic White (Predominantly first-year students)	Social networks (peer nominations and reciprocal ties within a residence hall)	Self-reported exercise hours per week	Gender, race/ethnicity, year in school, athlete status,interest in joining a fraternity/sorority.	The study found that peer exercise behavior within dormitory social networks was not significantly associated with individual exercise behavior.
(Deliens et al., 2015) [13]	Qualitative study using focus group discussions	46 university students (29F/17M, Mage = 20.7±1.6,AR = 18–26)	Brussels, Belgium	Belgian (no first year students)	Social support, parental control, peer influence	Self-reported physical activity, active transportation	Na	Parental control (such as time management) and peer influence (such as active participation in sports) within the social environment were identified as key factors influencing students’ physical activity and sedentary behavior.
(Harmon et al., 2016) [39]	Cross-sectional	N = 40,(26F/14M, Mage = 25.4± 7.9, AR≥18 )	Hawaii, America	predominantly Asian American or Mixed Ethnicity	Social networks (network nominations)	Meets recommendations MVPA (≥30 min/day)	Individual level: gender, age, ethnicity, screen time, percentage of fat intake, daily fruit and vegetable intake	The study indicated no significant association between social networks and physical activity (OR = 1.09, 95% CI 0.90–1.31).
(Scarapicchia et al., 2017) [52]	Longitudinal	819 first-year university students, (64%F/36%M, Mage = 18.28 ± 1.35)	University of Toronto, Canada	Predominantly Canadian, with significant Asian descent population	Social support networks, functional social support (emotional, esteem, tangible)	Moderate-to-vigorous physical activity (MVPA)	Gender, ethnicity, living situation, relationship status	The study showed that family social support had a significant positive impact on physical activity (β = 29.76, p<0.001), while support from friends was not significant.
(Klaiber et al., 2018) [53]	Longitudinal	67 during their first term, (74.6%/25.4%MF, Mage = 20.63 ± 1.35, AR = 17–24)	University of British Columbia, Canada	Predominantly Canadian, diverse international representation	Number of close friends (social integration), Perceived social support	Self-reported minutes of moderate-intensity physical activity per week	Gender, age, relocation to attend university, social anxiety, depressive symptoms	The study suggested a positive trend between making more friends and physical activity levels during university, but the association was not statistically significant.
(Bartshe et al., 2018) [40]	Cross-sectional	N = 410 (77.1%F/22.9%M, Mage = 24;AR = 18 ~ 61)	University of Nevada, Las Vegas, America	Mostly Caucasian	Social Capital(7 items social cohesion; 2 items trust; 16 items social participation)	Physical activity (recommendation of 150 min per week)	Individual level: age, gender, and owned a vehicle	The study found a significant positive association between social capital and physical activity (OR = 1.25, p = 0.04).
(Gesualdo & Pinquart, 2021) [54]	Cross-sectional	208 participants, (82.7%F/17.3%M, Mage = 20 .9± 4.1, AR≥18)	Philipps-University Marburg, Germany	During COVID-19, predominantly German	Health behaviors of close social ties (parents, partners, peers)	Self-reported physical activity frequency per week	Age, gender, whether the hometown is in Germany or abroad	The study showed that parental, partner, and peer support had a significant influence on physical activity frequency, with parents (r = 0.21, p = 0.01), partners (r = 0.31, p = 0.01), and peers (r = 0.31, p<0.001) having positive effects.
(Bartshe et al., 2023) [41]	Cross-sectiona	N = 403,(73.6%F/26.4%M, Mage = 24 ±7.42, AR≥18)	Las Vegas, Southern Nevada, America	Predominantly diverse	Social capital(social cohesion, social participation, trust, informal social contro)	Physical activity (met physical activity recommendations)	Na	The findings indicated a significant positive association between social capital and physical activity (OR = 1.308, p = 0.01).
(Li & Meng, 2023) [55]	Cross-sectional	328 participants, (62%F/38%M, Mage = 21.04±3.21)	Large Midwestern university, USA	Predominantly White	Communication within core and acquaintance networks, social control, awareness of healthy behaviors	Self-reported physical activity duration and frequency (moderate-to-vigorous physical activity)	Gender, age, education, stage of behavior change, attitude towards exercise	The study found that awareness of healthy behaviors within the core network was significantly associated with increased physical activity (β = 0.28, p<0.01). However, neither positive nor negative social control from the core or acquaintance networks had a significant effect on physical activity.
(Prochnow et al., 2023) [42]	Cross-sectiona	N = 250,(62.8%F/37.2%M, Mage = 20.25.±1.65, AR≥18)	Southern America	Predominantly White	Social networks developed through intramural sports	physical activity (meeting physical activity recommendations.)	Na	The study found that social networks formed through intramural sports were significantly associated with physical activity (OR = 2.47, p = 0.03), though the closeness of the network did not show a significant effect.

Note. M = Male, F = Female, Mage = Mean Age, AR = Age Range, Na = Not available.

### 3.3. Study quality assessment

Based on the quality assessment, all studies included in this review were rated as high quality (see Tables 3 and 4). All quantitative studies scored 85% or above, with the majority (8 out of 9) scoring between 95% and 100%, indicating strong study designs, appropriate sampling methods, and comprehensive reporting of results. The single qualitative study also received a high score, particularly due to the depth of data analysis and its strong alignment with theoretical frameworks. Overall, the evidence from these studies is considered reliable and supports the conclusions of this systematic review.

**Table 3:** Quality assessment of included quantitative studies.

Article	Quality Assessment Criteria														Total Score	Quality Score
	1	2	3	4	5	6	7	8	9	10	11	12	13	14		
(Barnett et al., 2014)[51]	Y	Y	Y	Y	n/a	n/a	n/a	Y	Y	Y	Y	Y	Y	Y	22	100%
(Harmon et al., 2016)[39]	Y	Y	Y	Y	n/a	n/a	n/a	Y	P	Y	P	P	Y	Y	19	85%
(Scarapicchia et al., 2017)[52]	Y	Y	Y	Y	n/a	n/a	n/a	Y	P	Y	Y	Y	Y	Y	21	95%
(Klaiber et al., 2018)[53]	Y	Y	Y	Y	n/a	n/a	n/a	Y	P	Y	Y	Y	Y	Y	21	95%
(Bartshe et al., 2018)[40]	Y	Y	Y	Y	n/a	n/a	n/a	Y	P	Y	Y	Y	Y	Y	21	95%
(Gesualdo & Pinquart, 2021)[54]	Y	Y	Y	Y	n/a	n/a	n/a	Y	Y	Y	Y	P	Y	Y	21	95%
(Bartshe et al., 2023)[41]	Y	Y	Y	Y	n/a	n/a	n/a	Y	Y	Y	Y	P	Y	Y	21	95%
(Li & Meng, 2023)[55]	Y	Y	Y	Y	n/a	n/a	n/a	Y	P	Y	Y	Y	Y	Y	21	95%
(Prochnow et al., 2023)[42]	Y	Y	Y	Y	n/a	n/a	n/a	Y	P	Y	Y	Y	Y	Y	21	95%

Note. 1) Question/objective sufficiently described? 2) Study design evident and appropriate? 3) Method of subject/comparison group selection or source of information/input variables described as appropriate? 4) Subject (and comparison group, if applicable) characteristics sufficiently described? 5) If interventional and random allocation was possible, was it described? 6) If interventional and blinding of investigators was possible, was it reported? 7) If interventional and blinding of subjects was possible, was it reported? 8) Outcome and (if applicable) exposure measure(s) well defined and robust to measurement/misclassification bias? means of assessment reported? 9) Sample size appropriate? 10) Analytical methods described/justified and appropriate? 11) Some estimate of variance is reported for the main results? 12) Controlled for confounding? 13) Results reported in sufficient detail? 14) Conclusions support the by results? Y = yes, P = partial, N = no, n/a = not applicable.

**Table 4:** Quality assessment of included qualitative studies.

Article	Quality Assessment Criteria										Total Score	Quality Score
	1	2	3	4	5	6	7	8	9	10		
(Deliens et al., 2015)[13]	Y	Y	Y	Y	Y	Y	Y	Y	Y	P	19	95%

Note. 1) Question/objective sufficiently described? 2) Study design evident and appropriate? 3) Context for the study clear? 4) Connection to a theoretical framework/wider body of knowledge? 5) Sampling strategy described, relevant, and justified? 6) Data collection method clearly described and systematic? 7) Data analysis clearly described and systematic? 8) Use of verification procedure(s) to establish credibility? 9) Conclusions supported the by results? 10) Reflexivity of the account? Y = yes, P = partial, N = no.

### 3.4. Network/Support-based social capital measures and physical activity

A total of eight studies examined the relationship between network/support-based measures of social capital and physical activity [51,13,54,39,52,53,55,42]. These studies suggest that social networks and social support may play a role in influencing physical activity among college and university students. Social networks are often considered a potential factor in promoting physical activity. Barnett et al. [51] investigated peer reciprocal relationships within college dormitories and found that these social networks were somewhat linked to individual exercise behaviors, although the results were not always statistically significant. This suggests that peer interactions may influence physical activity in certain contexts, but the impact may be moderated by factors such as individual motivation and interest. Gesualdo & Pinquart [54] further noted that health behaviors within close social relationships—such as those with parents, partners, and peers—were associated with students’ physical activity frequency, indicating that close relationships may play a role in the transmission of healthy behaviors. Prochnow et al. [42] explored social networks formed through campus-based sports activities and reported that these networks were associated with an increased likelihood of participants meeting recommended physical activity levels. This highlights the potential importance of sports-related social networks in supporting physical activity among college and university students.

Although Harmon et al. [39] did not find a statistically significant relationship between the number of social network connections and physical activity, their results suggested that social connections may have a potential relevance in shaping individual health behaviors. Similarly, Klaiber et al. [53] studied the relationship between the number of new friends made during the first semester of university and later physical activity levels. While they observed a positive trend suggesting that making more friends might be associated with higher physical activity levels, the association did not reach statistical significance, indicating that social integration may be linked to student physical activity but could be moderated by other factors. Additionally, Li & Meng [55] reported a significant positive association between health behavior awareness within core social networks and physical activity levels, suggesting that maintaining a healthy lifestyle among close friends or family may be linked to increased physical activity. However, the study also found that social control itself was not significantly associated with physical activity, indicating that external behavioral control may not be sufficient to significantly increase physical activity; instead, health behavior awareness formed through role modeling and support may be more influential.

Similarly, social support, particularly from family and friends, is also associated with increased physical activity. Scarapicchia et al. [52] found that emotional, esteem, and tangible support provided by family members were significantly associated with higher levels of MVPA, with support from friends also showing positive associations in certain contexts. Deliens et al. [13] conducted a qualitative study exploring the potential influence of parental control and peer influence on physical activity and sedentary behaviors among university students, particularly in relation to active transportation behaviors such as walking and cycling.

Overall, the current evidence suggests that the potential impact of social networks and social support on physical activity may depend on the quality and closeness of these relationships. Key peers, family members, and close friends seem to play a crucial role in providing support and modeling healthy behaviors, which may contribute to higher levels of physical activity among college and university students.

### 3.5. Social cohesion-based social capital measures and physical activity

A total of two studies examined the relationship between social cohesion-based measures of social capital and physical activity [40,41]. These studies suggest that social capital, as measured through social cohesion, may be an important factor in promoting physical activity. Bartshe et al. [40] assessed the potential impact of social cohesion, trust, and social participation on physical activity among university students. The results indicated that students with higher levels of social capital were more likely to meet the recommended physical activity levels. In a subsequent study, Bartshe et al. [41] incorporated informal social control, further supporting the association between higher social capital and increased physical activity. Overall, these two studies suggest that social cohesion-based dimensions of social capital may be relevant factors in encouraging physical activity among university students, highlighting the potential importance of community support and connectivity in increasing physical activity levels.

## 4. Discussion

This systematic review explored the relationship between different dimensions of social capital—such as social networks, support, and social cohesion—and physical activity among college and university students. The majority of the studies reviewed suggest that social capital may play a role in influencing physical activity in this population. Specifically, stronger social networks, support systems from family and friends, and higher levels of social cohesion were generally associated with increased physical activity. Moreover, the findings indicate that social capital may influence physical activity among college and university students through various mechanisms, particularly in group settings. However, since only ten studies were included in this review, these findings should be considered preliminary, and further research is needed to confirm these associations.

### 4.1. Network/Support-based social capital measures and physical activity

Social networks refer to the social relationships and connections individuals have with family, friends, colleagues, and neighbors, which can be measured through various characteristics such as size, density, relationship quality, and composition [30]. Social support, on the other hand, refers to the emotional, informational, and practical assistance obtained from these social networks [56]. Together, these reflect the impact of social capital from individual-level attributes [29]. This systematic review found that most forms of network/support-based social capital were positively associated with physical activity among college and university students [13,42,51,52,54,55]. The potential impact of these forms of social capital on physical activity may depend on the quality and closeness of the relationships. Specifically, college students’ social networks typically consist of close friends, teachers, classmates, roommates, family members, and, in some cases, teammates or members of extracurricular groups [57,58]. These networks are influenced by both academic and social environments, with frequent interactions occurring in dormitories, classrooms, sports teams, and other campus activities [59,60]. The quality and closeness of relationships are often reflected in the frequency of social interactions, the strength of trust, emotional support, and the practical help individuals receive from these relationships [61–63]. Previous research has shown that social networks exhibit characteristics of homophily, meaning that individuals tend to associate with others who share similar behaviors [64,65]. This is particularly relevant to physical activity, as less active individuals are more likely to form connections with others who also exhibit lower levels of activity [37]. However, the effectiveness of network-based social capital in promoting physical activity may depend not only on the quality and closeness of relationships but also on the activity levels within those networks. High-quality relationships are likely to promote physical activity only if the individuals within the network are themselves physically active [42,54,55]. This highlights the importance of network composition—the specific behaviors and norms prevalent within a social network—which may be a key factor in determining whether social capital can successfully promote physical activity. Further research is needed to investigate how social network analysis (SNA) can explore the detailed composition of student networks, particularly the distribution of different behavioral types within networks, and how this influences the role of social capital in promoting physical activity.

Moreover, these relationships may influence individual physical activity behaviors through various mechanisms, including role modeling, social norms, emotional support, information dissemination, and practical assistance. The findings of this review suggest that behavioral modeling and imitation within social networks may be one of the mechanisms influencing physical activity among college and university students. Role modeling refers to the phenomenon where individuals observe their friends or family members actively participating in physical activity, providing them with a behavioral model that may encourage them to imitate these actions and internalize them into their own behavior patterns [66]. For example, Li & Meng [55] found a significant association between health behavior awareness within core social networks and physical activity. This suggests that when close friends or family members maintain a healthy lifestyle, individuals are more likely to imitate these behaviors, thereby increasing their own physical activity levels. Prochnow et al. [42] further explored the impact of social networks formed through on-campus physical activities and found that students participating in these activities not only developed strong social connections but were also more likely to meet recommended levels of physical activity. This indicates that role modeling within social networks, through mechanisms of imitation and interaction, may play a role in promoting physical activity among college and university students. Deliens et al. [13] emphasized in their qualitative study that behavioral modeling within social networks may not only influence physical activity but also be linked to sedentary behavior patterns among students. However, it is important to note that Barnett et al. [51] found that peer interactions and behavioral modeling were somewhat related to individual exercise behaviors, but their influence may vary depending on individual motivation and interest.

Social norms define the behaviors that are accepted or expected within a group, and these norms can influence individual behavior choices to some extent [67]. Although social norms were not systematically explored as an independent variable in the studies included in this review, their potential influence appears to be reflected indirectly through other social factors. For example, the studies by Barnett et al. [51] and Li & Meng [55], while primarily focused on peer relationships and interpersonal interactions, indirectly support the potential role of social norms in shaping group behavior. Previous research suggests that the influence of social norms on group behavior may partly stem from social identity, where self-categorization plays a role in adhering to group norms, as well as from the individual’s need to integrate into the group and the motivation to guide their own behavior [68,69]. This implies that when the majority of members in a social network participate in a certain healthy behavior, this behavior can become a "default norm" within the group [70], subtly influencing other members to follow these norms to avoid being seen as disconnected or non-conforming. Although these studies did not explicitly address social norms, it can be inferred that the spread of group behavior may partly benefit from implicit social norms. Prochnow et al. [42] noted that through participation in on-campus physical activities, students formed strong social networks and a sense of collective involvement, which may have encouraged individuals to engage in more physical activity through the influence of social norms. Similarly, Harmon et al. [39] investigated social influence within networks and found that when students perceived their friends or peers to value physical activity, they were more likely to adopt the behavior themselves. However, this influence was not always strong, as it did not significantly predict whether individuals would meet MVPA recommendations.

Although this was not the primary focus of this systematic review, social support has consistently played an important role in promoting physical activity [43,71]. College students are at a critical turning point in life, and external support may have a profound impact on their behavioral choices [72]. Among the types of support, emotional support—by offering care, encouragement, and understanding—can enhance an individual’s self-efficacy, giving them greater confidence and motivation to engage in and maintain physical activity [36]. Scarapicchia et al. [52] found that emotional support from family and friends was significantly associated with moderate to vigorous physical activity, suggesting that individuals who receive emotional support are more likely to maintain higher levels of physical activity. Informational support, which includes providing relevant information about healthy lifestyles, exercise techniques, or opportunities for participation, can help individuals better understand how to engage in and sustain physical activity [73]. Previous research has shown that health information shared through social networks may enable individuals to access more resources and options, potentially reducing the barriers to participation in physical activity [74,75]. Practical assistance refers to the tangible support provided in everyday life [76], such as accompanying someone to exercise, creating workout plans, or providing resources for participation. This type of support can help college students overcome barriers to physical activity at a practical level, ensuring that they can consistently engage in healthy activities [44]. Social networks refer to an individual’s social relationships and connections, including those with family, friends, colleagues, and neighbors, and can be measured by various characteristics such as size, density, relationship quality, and composition [43]. Social support, on the other hand, refers to the emotional, informational, and practical assistance obtained from these social networks [44]. This systematic review found that most forms of network/support-based social capital have a significant positive effect on college and university students’ physical activity [7,29,38–40,42]. The effectiveness of these social capital forms in promoting physical activity depends on the quality and closeness of the relationships. They may influence individual physical activity behaviors through various mechanisms, including role modeling, social norms, emotional support, information dissemination, and practical assistance.

### 4.2. Social cohesion-based social capital and physical activity

Although the number of studies included in this review was limited, social capital measured by social cohesion is considered a potentially important factor influencing physical activity among college and university students [40,41]. The findings of this review are generally consistent with the existing literature, further supporting the potential significance of social cohesion in promoting healthy behaviors [34,77,78]. Previous research has also shown that social cohesion can foster cooperation and participation within groups, thereby increasing the frequency of health behaviors [79]. Social cohesion refers to the trust, sense of belonging, mutual cooperation, and participation among members of a group, which together contribute to a collective sense of unity and solidarity within social networks [33]. Higher levels of social cohesion are often associated with greater collective participation, closer social relationships, and a stronger sense of belonging for individuals [80]. These elements provide potential mechanisms through which social cohesion may promote physical activity.

Firstly, social cohesion may enhance individuals’ sense of collective participation, encouraging college students to engage more actively in group activities [81,82]. This sense of participation may increase opportunities for physical activity and improve the quality of such activities [83]. Secondly, high levels of social cohesion are typically accompanied by stronger social support networks [84]. These networks within a community or group may provide more robust emotional support, informational support, and practical assistance, thereby boosting individuals’ motivation to engage in physical activity. Furthermore, social cohesion is closely tied to a sense of belonging and identity among college students [85]. This sense of belonging can be understood as students’ perception of social support on campus, which is also considered a key factor for academic success [86]. A strong sense of belonging may not only encourage students to participate in group activities but also enhance their persistence in such activities [87].

### 4.3. Limitations

It is important to acknowledge some limitations of the existing evidence base. Many studies included in this review relied on self-reported data, which may introduce bias due to inaccuracies or social desirability responses. Additionally, most of the included studies were cross-sectional, making it difficult to draw strong causal inferences about the relationship between social capital and physical activity. These limitations are inherent to the primary studies and reflect the challenges of accurately measuring social capital and physical activity behaviors.

As for the limitations of this review itself, the number of studies included was relatively small—especially those examining specific dimensions of social capital (such as social cohesion) or focusing on different student subgroups—which may limit the generalizability of the findings. Furthermore, most of these studies concentrated on college students in Western countries, introducing potential cultural and geographical biases that limit the applicability of the conclusions to non-Western populations. Addition0ally, the studies included in this review did not clearly specify or differentiate the timeframes or locations of physical activity (e.g., on-campus vs. off-campus, during semesters or breaks), which limits our ability to explore the dynamic relationship between different social capital indicators and physical activity under varying conditions. This lack of distinction may obscure the specific roles of different types of social support or social cohesion, depending on where students primarily reside during their studies. Finally, this review predominantly included studies published in English, which may have overlooked relevant research in other languages, particularly from non-English-speaking countries, further restricting the scope and generalizability of the findings.

### 4.4. Future directions

To improve the accuracy of future research, more objective measurement tools, such as accelerometers and activity trackers, should be utilized to reduce self-reporting bias and provide more reliable data on physical activity. Longitudinal study designs should also be prioritized to better understand the causal relationships and long-term interactive effects of social capital on physical activity. Given the unique context of students, social cohesion within the school environment could be a potential focus for future research [83]. Examining how social cohesion within academic settings influences physical activity, and how positive interpersonal relationships, group solidarity, and a sense of belonging on campus promote students’ physical activity, will provide valuable insights.

In addition, a systems perspective could enhance our understanding of how social capital operates across multiple levels of influence, including individual, social, environmental, and policy factors [88]. Social network analysis (SNA) offers a powerful tool for uncovering the dynamics of social capital within student populations [31]. By adopting SNA, future research can explore the structural characteristics of social networks (such as network density, centrality, and reciprocity) and their roles in promoting or inhibiting physical activity. These analyses can reveal how different configurations of social networks (e.g., active or inactive groups) influence students’ participation in physical activity. A systems approach would also consider how these social networks interact with broader environmental and institutional factors, providing a more comprehensive understanding of the underlying mechanisms.

Future research should also investigate the specific mechanisms through which social capital influences physical activity, including emotional support, role modeling, and social norms. A systems-based approach can elucidate how these mechanisms operate in different environments and among various populations. Understanding how social capital functions under different conditions—such as varying social backgrounds, ethnicities, genders, and economic statuses—will be key to uncovering the mediating mechanisms and the interactive effects on physical activity.

Another important area for future research is the potential negative effects of social capital [89]. While social capital is generally viewed as a positive force for promoting health behaviors, in certain cases, it may lead to exclusionary behaviors or peer pressure [90], which could have adverse effects on physical activity. Exploring these negative aspects will help us develop a more nuanced understanding of the complexities of social capital and provide insights for designing balanced and effective health interventions.

Finally, in terms of practical implications, while this study highlights the potential of social capital in promoting physical activity, it is crucial to translate these findings into actionable interventions. For example, universities could implement programs designed to intentionally foster social networks, such as peer mentoring programs or student groups centered around physical activity. These initiatives could help build strong support networks that encourage students to be more active. Additionally, interventions could target key social influencers within student networks, leveraging their influence to promote and sustain physical activity behaviors across the broader student population.

## 5. Conclusions

This systematic review suggests that social capital may play a role in promoting physical activity among college and university students, particularly through strong social networks, support from family and friends, and high levels of social cohesion. These findings imply that social capital may influence physical activity in students through various mechanisms, especially in group settings. Strengthening these elements of social capital could potentially help increase physical activity levels among students, thereby improving their overall health. However, the reliance on self-reported data and the predominance of cross-sectional studies limit the ability to draw strong causal conclusions, and the lack of clarity regarding the context and timing of physical activity in the reviewed studies hinders understanding of how different social capital indicators impact activity under varying conditions. Future research should employ objective measurement tools and longitudinal designs to better capture these dynamics. Universities could also consider interventions like peer mentoring and student activity groups, utilizing key social influencers to support and sustain physical activity within the student community.

## Supporting information

S1 Checklist PRISMA 2020 checklist. (DOCX)

S1 File Complete search, screen and extract data process. (XLSX)

## References

[1] Ding D, Ramirez Varela A, Bauman AE, Ekelund U, Lee I-M, Heath G, et al. Towards better evidence-informed global action: lessons learnt from the Lancet series and recent developments in physical activity and public health. Br J Sports Med. 2020;54: 462–468. doi: 10.1136/bjsports-2019-101001 31562122 PMC7146932

[2] Mahindru A, Patil P, Agrawal V. Role of physical activity on mental health and well-being: A review. Cureus. 2023;15: e33475. doi: 10.7759/cureus.33475 36756008 PMC9902068

[3] Mandolesi L, Polverino A, Montuori S, Foti F, Ferraioli G, Sorrentino P, et al. Effects of Physical Exercise on Cognitive Functioning and Wellbeing: Biological and Psychological Benefits. Front Psychol. 2018;9: 509. doi: 10.3389/fpsyg.2018.00509 29755380 PMC5934999

[4] Bidzan-Bluma I, Lipowska M. Physical Activity and Cognitive Functioning of Children: A Systematic Review. Int J Environ Res Public Health. 2018;15: 800. doi: 10.3390/ijerph15040800 29671803 PMC5923842

[5] Marquez DX, Aguiñaga S, Vásquez PM, Conroy DE, Erickson KI, Hillman C, et al. A systematic review of physical activity and quality of life and well-being. Transl Behav Med. 2020;10: 1098–1109. doi: 10.1093/tbm/ibz198 33044541 PMC7752999

[6] Maselli M, Ward PB, Gobbi E, Carraro A. Promoting Physical Activity Among University Students: A Systematic Review of Controlled Trials. Am J Health Promot. 2018;32: 1602–1612. doi: 10.1177/0890117117753798 29366334

[7] Kljajević V, Stanković M, Đorđević D, Trkulja-Petković D, Jovanović R, Plazibat K, et al. Physical Activity and Physical Fitness among University Students—A Systematic Review. Int J Environ Res Public Health. 2021;19: 158. doi: 10.3390/ijerph19010158 35010418 PMC8750240

[8] Yuan F, Peng S, Khairani AZ, Liang J. A Systematic Review and Meta-Analysis of the Efficacy of Physical Activity Interventions among University Students. Sustainability. 2024;16: 1369. doi: 10.3390/su16041369

[9] Hirvensalo M, Lintunen T. Life-course perspective for physical activity and sports participation. Eur Rev Aging Phys Act. 2011;8: 13–22. doi: 10.1007/s11556-010-0076-3

[10] Lederer AM, Oswalt SB. The value of college health promotion: a critical population and setting for improving the public’s health. Am J Health Educ. 2017;48: 215–218. doi: 10.1080/19325037.2017.1316692

[11] Almutairi KM, Alonazi WB, Vinluan JM, Almigbal TH, Batais MA, Alodhayani AA, et al. Health promoting lifestyle of university students in Saudi Arabia: a cross-sectional assessment. BMC Public Health. 2018;18: 1093. doi: 10.1186/s12889-018-5999-z 30185167 PMC6126031

[12] Bennasar-Veny M, Yañez AM, Pericas J, Ballester L, Fernandez-Dominguez JC, Tauler P, et al. Cluster Analysis of Health-Related Lifestyles in University Students. Int J Environ Res Public Health. 2020;17: 1776. doi: 10.3390/ijerph17051776 32182922 PMC7084566

[13] Deliens T, Deforche B, De Bourdeaudhuij I, Clarys P. Determinants of physical activity and sedentary behaviour in university students: a qualitative study using focus group discussions. BMC Public Health. 2015;15: 201. doi: 10.1186/s12889-015-1553-4 25881120 PMC4349731

[14] Anjali, Sabharwal M. Perceived Barriers of Young Adults for Participation in Physical Activity. Current Research in Nutrition and Food Science Journal. 2018;6: 437–449.

[15] Wengreen HJ, Moncur C. Change in diet, physical activity, and body weight among young-adults during the transition from high school to college. Nutr J. 2009;8: 32. doi: 10.1186/1475-2891-8-32 19624820 PMC2720988

[16] Kwan MY, Cairney J, Faulkner GE, Pullenayegum EE. Physical Activity and Other Health-Risk Behaviors During the Transition Into Early Adulthood: A Longitudinal Cohort Study. American Journal of Preventive Medicine. 2012;42: 14–20. doi: 10.1016/j.amepre.2011.08.026 22176841

[17] Deforche B, Van Dyck D, Deliens T, De Bourdeaudhuij I. Changes in weight, physical activity, sedentary behaviour and dietary intake during the transition to higher education: a prospective study. International Journal of Behavioral Nutrition and Physical Activity. 2015;12: 16. doi: 10.1186/s12966-015-0173-9 25881147 PMC4332914

[18] Winpenny EM, Smith M, Penney T, Foubister C, Guagliano JM, Love R, et al. Changes in physical activity, diet, and body weight across the education and employment transitions of early adulthood: A systematic review and meta-analysis. Obesity Reviews. 2020;21: e12962. doi: 10.1111/obr.12962 31955496 PMC7079102

[19] Corder K, Winpenny E, Love R, Brown HE, White M, Sluijs E van. Change in physical activity from adolescence to early adulthood: a systematic review and meta-analysis of longitudinal cohort studies. Br J Sports Med. 2019;53: 496–503. doi: 10.1136/bjsports-2016-097330 28739834 PMC6250429

[20] Ferreira Silva RM, Mendonça CR, Noll M. Barriers to high school and university students’ physical activity: A systematic review protocol. International Journal of Educational Research. 2021;106: 101743. doi: 10.1016/j.ijer.2021.101743 PMC8979430 35377905

[21] Brown CEB, Richardson K, Halil-Pizzirani B, Atkins L, Yücel M, Segrave RA. Key influences on university students’ physical activity: a systematic review using the Theoretical Domains Framework and the COM-B model of human behaviour. BMC Public Health. 2024;24: 418. doi: 10.1186/s12889-023-17621-4 38336748 PMC10854129

[22] Gil-Espinosa FJ, Nielsen-Rodríguez A, Romance R, Burgueño R. Smartphone applications for physical activity promotion from physical education. Educ Inf Technol (Dordr). 2022;27: 11759–11779. doi: 10.1007/s10639-022-11108-2 35610980 PMC9118821

[23] Lindström M. Social capital, desire to increase physical activity and leisure-time physical activity: A population-based study. Public health. 2011;125: 442–7. doi: 10.1016/j.puhe.2011.01.015 21771550

[24] Chen W-L, Zhang C-G, Cui Z-Y, Wang J-Y, Zhao J, Wang J-W, et al. The impact of social capital on physical activity and nutrition in China: the mediating effect of health literacy. BMC Public Health. 2019;19: 1713. doi: 10.1186/s12889-019-8037-x 31856789 PMC6924071

[25] Aliyas Z. Social Capital and Physical Activity Level in an Urban Adult Population. American Journal of Health Education. 2020;51: 40–49. doi: 10.1080/19325037.2019.1691092

[26] Bhandari H, Yasunobu K. What Is Social Capital? A Comprehensive Review of the Concept. Asian Journal of Social Science. 2009;37: 480–510. doi: 10.1163/156853109X436847

[27] Murayama H, Fujiwara Y, Kawachi I. Social Capital and Health: A Review of Prospective Multilevel Studies. Journal of Epidemiology. 2012;22: 179–187. doi: 10.2188/jea.je20110128 22447212 PMC3798618

[28] Villalonga-Olives E, Kawachi I. The measurement of social capital. Gaceta Sanitaria. 2015;29: 62–64. doi: 10.1016/j.gaceta.2014.09.006 25444390

[29] Kawachi I, Subramanian SV, Kim D. Social Capital and Health. In: Kawachi I, Subramanian SV, Kim D, editors. Social Capital and Health. New York, NY: Springer; 2008. pp. 1–26. doi: 10.1007/978-0-387-71311-3_1

[30] Li N, Huang Q, Ge X, He M, Cui S, Huang P, et al. A Review of the Research Progress of Social Network Structure. Complexity. 2021;2021: e6692210. doi: 10.1155/2021/6692210

[31] Tabassum S, Pereira F, Fernandes S, Gama J. Social network analysis: An overview. Wiley Interdisciplinary Reviews: Data Mining and Knowledge Discovery. 2018;8: e1256. doi: 10.1002/widm.1256

[32] Drageset J. Social support. Health Promotion in Health Care–Vital Theories and Research [Internet]. Springer; 2021. doi: 10.1007/978-3-030-63135-2_11 36315659

[33] Moustakas L. Social Cohesion: Definitions, Causes and Consequences. Encyclopedia. 2023;3: 1028–1037. doi: 10.3390/encyclopedia3030075

[34] Rodgers J, Valuev AV, Hswen Y, Subramanian SV. Social capital and physical health: An updated review of the literature for 2007–2018. Social Science & Medicine. 2019;236: 112360. doi: 10.1016/j.socscimed.2019.112360 31352315

[35] Xue X, Reed WR, Menclova A. Social capital and health: a meta-analysis. Journal of Health Economics. 2020;72: 102317. doi: 10.1016/j.jhealeco.2020.102317 32497954

[36] Zhang Y, Hasibagen, Zhang C. The influence of social support on the physical exercise behavior of college students: The mediating role of self-efficacy. Frontiers in Psychology. 2022;13. Available: https://www.frontiersin.org/articles/10.3389/fpsyg.2022.1037518. doi: 10.3389/fpsyg.2022.1037518 36532973 PMC9756807

[37] Mötteli S, Dohle S. Egocentric social network correlates of physical activity. J Sport Health Sci. 2020;9: 339–344. doi: 10.1016/j.jshs.2017.01.002 32768126 PMC7411096

[38] Tsoli S, Fancourt D, Sullivan A, Hamer M, Ploubidis GB, Kawachi I. Life-course social participation and physical activity in midlife: longitudinal associations in the 1970 British Cohort Study (BCS70). Eur J Epidemiol. 2024;39: 643–651. doi: 10.1007/s10654-024-01107-7 38492116 PMC11249713

[39] Harmon BE, Forthofer M, Bantum EO, Nigg CR. Perceived influence and college students’ diet and physical activity behaviors: an examination of ego-centric social networks. BMC Public Health. 2016;16: 473. doi: 10.1186/s12889-016-3166-y 27267371 PMC4895992

[40] Bartshe M, Coughenour C, Pharr J. Perceived Walkability, Social Capital, and Self-Reported Physical Activity in Las Vegas College Students. Sustainability. 2018;10. doi: 10.3390/su10093023

[41] Bartshe M, Coughenour C, Stephen H. The relationship between tree canopy and social capital on physical activity in college students. Journal of American College Health. 2023;71: 1705–1714. doi: 10.1080/07448481.2021.1947299 34314667

[42] Prochnow T, Park JH, Patterson MS. Intramural sports social networks and implications for college student physical activity, sense of community, and retention. Journal of American College Health. 2023. doi: 10.1080/07448481.2023.2239367 37531217

[43] Scarapicchia TMF, Amireault S, Faulkner G, Sabiston CM. Social support and physical activity participation among healthy adults: a systematic review of prospective studies. International Review of Sport and Exercise Psychology. 2017;10: 50–83. doi: 10.1080/1750984X.2016.1183222

[44] Van Luchene P, Delens C. The Influence of Social Support Specific to Physical Activity on Physical Activity Among College and University Students: A Systematic Review. J Phys Act Health. 2021;18: 737–747. doi: 10.1123/jpah.2020-0713 33883289

[45] Prochnow T, Patterson MS. Assessing Social Network Influences on Adult Physical Activity Using Social Network Analysis: A Systematic Review. Am J Health Promot. 2022;36: 537–558. doi: 10.1177/08901171211060701 34898289

[46] Page MJ, McKenzie JE, Bossuyt PM, Boutron I, Hoffmann TC, Mulrow CD, et al. The PRISMA 2020 statement: An updated guideline for reporting systematic reviews. International Journal of Surgery. 2021;88: 105906. doi: 10.1016/j.ijsu.2021.105906 33789826

[47] Mendonça G, Cheng LA, Mélo EN, de Farias Júnior JC. Physical activity and social support in adolescents: a systematic review. Health Education Research. 2014;29: 822–839. doi: 10.1093/her/cyu017 24812148

[48] Wang X, Yang X, Juzaily Bin Mohd Nasiruddin N, Wei S, Dong D, Bin Samsudin S. Social Support and Physical Activity in College and University Students: A Meta-Analysis. Health Educ Behav. 2024; 10901981231216735. doi: 10.1177/10901981231216735 38305027

[49] Villalonga-Olives E, Wind TR, Kawachi I. Social capital interventions in public health: A systematic review. Social Science & Medicine. 2018;212: 203–218. doi: 10.1016/j.socscimed.2018.07.022 30048843

[50] Kmet LM. Standard quality assessment criteria for evaluating primary research papers from a variety of fields. Alberta Innovates—Health Solutions; 2004. Available: https://policycommons.net/artifacts/1222022/standard-quality-assessment-criteria-for-evaluating-primary-research-papers-from-a-variety-of-fields/1775098/.

[51] Barnett NP, Ott MQ, Rogers ML, Loxley M, Linkletter C, Clark MA. Peer Associations for Substance Use and Exercise in a College Student Social Network. HEALTH PSYCHOLOGY. 2014;33: 1134–1142. doi: 10.1037/a0034687 24364375

[52] Scarapicchia TMF, Sabiston CM, Pila E, Arbour-Nicitopoulos KP, Faulkner G. A longitudinal investigation of a multidimensional model of social support and physical activity over the first year of university. PSYCHOLOGY OF SPORT AND EXERCISE. 2017;31: 11–20. doi: 10.1016/j.psychsport.2017.03.011

[53] Klaiber P, Whillans AV, Chen FS. Long-Term Health Implications of Students’ Friendship Formation during the Transition to University. APPLIED PSYCHOLOGY-HEALTH AND WELL BEING. 2018;10: 290–308. doi: 10.1111/aphw.12131 29740963

[54] Gesualdo C, Pinquart M. Health behaviors of German university freshmen during COVID-19 in association with health behaviors of close social ties, living arrangement, and time spent with peers. Health Psychol Behav. 2021;9: 582–599. doi: 10.1080/21642850.2021.1947291 34285824 PMC8266231

[55] Li L, Meng JB. Network effects on physical activity through interpersonal vs. masspersonal communication with the core and acquaintance networks. COMPUTERS IN HUMAN BEHAVIOR. 2023;141. doi: 10.1016/j.chb.2022.107594

[56] Smith GSE, Moyle W, Burton NW. The Relationship between Social Support for Physical Activity and Physical Activity across Nine Years in Adults Aged 60–65 Years at Baseline. International Journal of Environmental Research and Public Health. 2023;20. doi: 10.3390/ijerph20054531 36901538 PMC10002128

[57] Mayer A, Puller SL. The old boy (and girl) network: social network formation on university campuses. J Public Econ. 2008;92: 329–347. doi: 10.1016/j.jpubeco.2007.09.001

[58] Pradeep Kumar P.C., Antony S, Murthy P, Thirumoorthy A, Philip M. Association of social network characteristics with substance use among college-going young adults: A cross-sectional study. Indian J Psychol Med. 2023;45: 155–161. doi: 10.1177/02537176221148971 36925503 PMC10011842

[59] Mayhew MJ, Rockenbach AB, Bowman NA, Seifert TA, Wolniak GC, Pascarella ET, et al. How college affects students: 21st century evidence that higher education works—university of iowa. 2016. Available: https://iro.uiowa.edu/esploro/outputs/book/How-college-affects-students-21st-century/9984285549002771.

[60] Zheng Z, Zeng M, Huang W, Sun N. The influence of university library environment on student interactions and college students’ learning engagement. Humanit Soc Sci Commun. 2024;11. doi: 10.1057/s41599-024-02892-y

[61] Reis H, FRANKS P. The role of intimacy and social support in health outcomes: Two processes or one? Personal Relationships. 2005;1: 185–197. doi: 10.1111/j.1475-6811.1994.tb00061.x

[62] Umberson D, Montez JK. Social Relationships and Health: A Flashpoint for Health Policy. J Health Soc Behav. 2010;51: S54–S66. doi: 10.1177/0022146510383501 20943583 PMC3150158

[63] Sutcliffe A, Binder J, Dunbar R. Activity in social media and intimacy in social relationships. Computers in Human Behavior. 2018;85. doi: 10.1016/j.chb.2018.03.050

[64] Lönnqvist J-E, Itkonen JVA. Homogeneity of personal values and personality traits in facebook social networks. Journal of Research in Personality. 2016;60: 24–35. doi: 10.1016/j.jrp.2015.11.001

[65] Mcpherson M, Smith-Lovin L, Cook J. Birds of a feather: Homophily in social networks. Annu Rev Sociol. 2001;27: 415–444. doi: 10.1146/annurev.soc.27.1.415

[66] Morgenroth T, Ryan MK, Peters K. The Motivational Theory of Role Modeling: How Role Models Influence Role Aspirants’ Goals. Review of General Psychology. 2015;19: 465–483. doi: 10.1037/gpr0000059

[67] Bicchieri C, Muldoon R, Sontuoso A. Social Norms. Winter 2023. In: Zalta EN, Nodelman U, editors. The Stanford Encyclopedia of Philosophy. Winter 2023. Metaphysics Research Lab, Stanford University; 2023. Available: https://plato.stanford.edu/archives/win2023/entries/social-norms/.

[68] Hogg MA, Reid SA. Social Identity, Self-Categorization, and the Communication of Group Norms. Communication Theory. 2006;16: 7–30. doi: 10.1111/j.1468-2885.2006.00003.x

[69] Hornsey MJ. Social Identity Theory and Self-categorization Theory: A Historical Review. Social and Personality Psychology Compass. 2008;2: 204–222. doi: 10.1111/j.1751-9004.2007.00066.x

[70] Dempsey RC, McAlaney J, Bewick BM. A Critical Appraisal of the Social Norms Approach as an Interventional Strategy for Health-Related Behavior and Attitude Change. Frontiers in Psychology. 2018;9. doi: 10.3389/fpsyg.2018.02180 30459694 PMC6232455

[71] Lin H, Chen H, Liu Q, Xu J, Li S. A meta-analysis of the relationship between social support and physical activity in adolescents: the mediating role of self-efficacy. Front Psychol. 2024;14. doi: 10.3389/fpsyg.2023.1305425 38282843 PMC10811609

[72] McLean L, Gaul D, Penco R. Perceived Social Support and Stress: a Study of 1st Year Students in Ireland. Int J Ment Health Addict. 2022; 1–21. doi: 10.1007/s11469-021-00710-z 35103049 PMC8791695

[73] García-Martín MÁ, Hombrados-Mendieta I, Gómez-Jacinto L. A Multidimensional Approach to Social Support: The Questionnaire on the Frequency of and Satisfaction with Social Support (QFSSS). Anales de Psicología / Annals of Psychology. 2016;32: 501–515. doi: 10.6018/analesps.32.2.201941

[74] Shi J, Poorisat T, Salmon CT. The Use of Social Networking Sites (SNSs) in Health Communication Campaigns: Review and Recommendations. Health Communication. 2018;33: 49–56. doi: 10.1080/10410236.2016.1242035 27858464

[75] Lee J, Turner K, Xie Z, Kadhim B, Hong Y-R. Association Between Health Information‒Seeking Behavior on YouTube and Physical Activity Among U.S. Adults: Results From Health Information Trends Survey 2020. AJPM Focus. 2022;1: 100035. doi: 10.1016/j.focus.2022.100035 37791235 PMC10546545

[76] Morelli SA, Lee IA, Arnn ME, Zaki J. Emotional and instrumental support provision interact to predict well-being. Emotion. 2015;15: 484–493. doi: 10.1037/emo0000084 26098734 PMC4516598

[77] Chuang Y-C, Chuang K-Y, Yang T-H. Social cohesion matters in health. Int J Equity Health. 2013;12: 87. doi: 10.1186/1475-9276-12-87 24165541 PMC4174898

[78] Miller HN, Thornton CP, Rodney T, Thorpe RJ, Allen J. Social Cohesion in Health. ANS Adv Nurs Sci. 2020;43: 375–390. doi: 10.1097/ANS.0000000000000327 32956090 PMC8344069

[79] Adetunji A, Silva M, Tulsiani NJ, Adediran M. “Like a broom tied together”: A qualitative exploration of social cohesion and its role in community capacity strengthening to support integrated health in Nigeria. PLOS Glob Public Health. 2023;3: e0002508. doi: 10.1371/journal.pgph.0002508 37874785 PMC10597522

[80] Schiefer D, Van der Noll J. The Essentials of Social Cohesion: A Literature Review. Social Indicators Research. 2017;132. doi: 10.1007/s11205-016-1314-5

[81] Thornton C, Miller P, Perry K. The impact of group cohesion on key success measures in higher education. Journal of Further and Higher Education. 2019;44: 1–12. doi: 10.1080/0309877X.2019.1594727

[82] Yoon P, Leem J. The Influence of Social Presence in Online Classes Using Virtual Conferencing: Relationships between Group Cohesion, Group Efficacy, and Academic Performance. Sustainability. 2021;13: 1988. doi: 10.3390/su13041988

[83] Veerman G-J, Denessen E. Social cohesion in schools: A non-systematic review of its conceptualization and instruments. Zhao Z, editor. Cogent Education. 2021;8: 1940633. doi: 10.1080/2331186X.2021.1940633

[84] Vonneilich N. Social Relations, Social Capital, and Social Networks: A Conceptual Classification. In: Klärner A, Gamper M, Keim-Klärner S, Moor I, von der Lippe H, Vonneilich N, editors. Social Networks and Health Inequalities: A New Perspective for Research. Cham: Springer International Publishing; 2022. pp. 23–34. doi: 10.1007/978-3-030-97722-1_2

[85] Clopton AW, Finch BL. Are college kids “bowling alone?” examining the contribution of team identification to the social capital of college students. Journal of Sport Behavior. 2010;33: 377–402.

[86] Strayhorn T. College Students’ Sense of Belonging. 2018. doi: 10.4324/9781315297293

[87] Gopalan M, Brady ST. College Students’ Sense of Belonging: A National Perspective. Educational Researcher. 2020;49: 134–137. doi: 10.3102/0013189x19897622 38847009 PMC11156233

[88] Nau T, Bauman A, Smith BJ, Bellew W. A scoping review of systems approaches for increasing physical activity in populations. Health Research Policy and Systems. 2022;20: 104. doi: 10.1186/s12961-022-00906-2 36175916 PMC9524093

[89] Baycan T, Öner Ö. The dark side of social capital: a contextual perspective. Ann Reg Sci. 2023;70: 779–798. doi: 10.1007/s00168-022-01112-2

[90] Villalonga-Olives E, Kawachi I. The dark side of social capital: A systematic review of the negative health effects of social capital. Social Science & Medicine. 2017;194: 105–127. doi: 10.1016/j.socscimed.2017.10.020 29100136

